# Navigating Challenging Conversations: The Interplay Between Inquiry and Knowledge Drives Preparation for Future Learning

**DOI:** 10.5334/pme.949

**Published:** 2023-07-28

**Authors:** Anne A. Kawamura, Leah Brown, Angela Orsino, Mohammad S. Zubairi, Maria Mylopoulos

**Affiliations:** 1 Department of Pediatrics, University of Toronto, Canada; 2 Bloorview Research Institute, Toronto, Ontario, Canada; 3 Wilson Centre for Research in Education, Toronto, Ontario, Canada; 4 Faculty of Medicine, University of Toronto, Canada; 5 Department of Pediatrics, McMaster University, Canada; 6 McMaster Education Research, Innovation, and Theory (MERIT) Program, McMaster University, Hamilton, Ontario, Canada

## Abstract

**Introduction:**

While some physicians hone their skills through informal learning in clinical practice, others do not. There is a lack of understanding of *why* some physicians seek improvement and *how* they use the workplace context to build their capabilities. Because physicians rarely pursue formal professional development activities to improve communication skills, examining physician-patient communication offers a powerful opportunity to illuminate important aspects of preparation for future learning in the workplace.

**Methods:**

This qualitative observational study involved over 100 hours of observation of eight pediatric rehabilitation physicians as they interacted with patients and families at an academic teaching hospital in 2018–2020. Detailed field notes of observations, post-observation interviews, and exit interviews were the data sources. Data collection and analysis using a constructivist grounded theory approach occurred iteratively, and themes were identified through constant comparative analysis.

**Results:**

Through their daily work, experienced physicians employ ‘habits of inquiry’ by constantly seeking a better understanding of how to navigate challenging conversations in practice through monitoring and attuning to situational and contextual cues, taking risks and navigating uncertainty while exploring new and varied ways of practicing, and seeking why their strategies are successful or not.

**Discussion:**

Engaging in communication challenges drives physician learning through an interplay between habits of inquiry and knowledge: inquiry into how to improve their communication supported by existing conceptual knowledge to generate new strategies. These ‘habits of inquiry’ prompt continual reinvestment in problem solving to refine existing knowledge and to build new skills for navigating communication challenges in practice.

## Introduction

Physicians who are noted for their exceptional diagnostic and professional practice, acquire and maintain knowledge and skills through purposeful, continuous learning in clinical practice [1,2,3,4]. They recognize when straightforward application of existing knowledge is insufficient and are able to generate new solutions [5]. That is, they demonstrate preparation for future learning (PFL): “the ability to learn new information, make effective use of resources, and invent new procedures in order to support learning and problem solving” [6] in their domain of work. PFL is critical to the theoretical framework of adaptive expertise as it explains how physicians balance their efficient use of existing knowledge with the need to innovate and generate new knowledge [7]. In addition to having acquired a rich network of procedural and conceptual knowledge that they are able put to flexible and generative use, there is mounting evidence that adaptive expert physicians who are prepared for future learning, continue to learn *through activities* in their daily clinical work [8,9,10]. Thus, physicians providing expert care continually innovate and build knowledge through their purposeful engagement in daily work activities.

However, what remains unknown are the *mechanisms* through which physicians actively learn from the workplace context. Previous research suggests that learning in the workplace requires both metacognitive skills and the ability to learn opportunistically [11]. For residents, feelings of competence and having the autonomy to regulate one’s own learning leads to improvements in performance and increases in time spent on learning [12]. Researchers, however, have suggested that learning in residency training may be different from active learning in the workplace for continuing professional development (CPD)[11]. Additionally, there is no clear understanding of how experienced physicians engage in learning as they provide adaptive expert care in the workplace.

In communication, a flexible and adaptive approach in response to challenging conversations is characteristic of adaptive expertise and requires PFL. Physicians must be able to elicit information and respond to patients in nuanced, empathic ways while providing patient-centred care. Challenging conversations involving conflict or intense emotion may especially require a dynamic, moment-to-moment monitoring of contextual cues and use of flexible and generative communication capabilities as the physician interacts with the patient [13,14,15]. Several studies have demonstrated that rich procedural knowledge (e.g. knowing *how*) complemented by an interconnected network of conceptual understanding (e.g. knowing *why*) supports this new learning, allowing flexible application of strategies in different situations and innovation for new or unexpected events [16,17,18,19,20], including communication [21].

While it’s clear that adaptive expert communication is crucial to providing patient-centred and compassionate care, rising patient complaints and increased litigation indicate that physician-patient communication remains problematic [22,23]. Moreover, physicians rarely seek formal professional development opportunities to develop their communication skills [24]. While some physicians hone their skills through informal learning in clinical practice by monitoring feedback cues from their daily work, others do not [25]. There is a lack of understanding of *why* some physicians seek improvement in their communication skills and *how* they use the workplace context to build their capabilities. Examining this more carefully may illuminate important aspects of PFL and improve how adaptive expertise is developed and enacted in the workplace. Accordingly, the purpose of this study was to explore how experienced physicians continue to improve their communication skills in the workplace context.

## Methods

We conducted an observational study with a constructivist grounded theory approach to data analysis. By using this methodology, we aimed to develop a substantive micro-level theory to explain how physicians engage in ongoing learning as they work, which is critical to PFL and adaptive expertise. This study extends previous work by the researchers, which have focused on the development of adaptive expertise and the mechanisms underlying adaptability and innovation central to this theoretical framework [4,21]. In addition, we brought our personal perspectives to the interpretation of the data as developmental pediatricians and educators who have been learning and teaching how to navigate challenging conversations in this context (AK, AO, MZ).Our insider preconceptions and experiences inevitably shaped how we viewed learning communication skills in the workplace. To remain open to new interpretations as the data was iteratively analyzed, we individually coded data before engaging in team dialogues and engaged outsider views from a medical student (LB) and education researcher (MM). We co-constructed theory by integrating participants’ perspectives with the team’s diverse clinical and educational experiences, as well as theoretical knowledge of adaptive expertise.

### Participants

We observed and interviewed eight experienced physicians (3 males, 5 females) from an academic tertiary care pediatric rehabilitation hospital in a large urban setting, who had been in practice for a minimum of five years. Our purposive and convenient sample consisted of experienced physicians practicing at this institution who were past the stage of transitioning to independent practice and have had opportunities to build their skills in communicating with patients and families over time. The theoretical framework of adaptive expertise does not take the essentialist view that individuals possess expert ‘skills’ and ‘traits’ that will be applied across situations. Instead, both the efficiency and innovation dimensions of adaptive expertise are better accounted for by contextual ‘states’ [26] in that their performance is driven by the immediate problem solving demands [27,28]. Thus, rather than identifying individuals defined as adaptive experts, our sampling strategy allowed us to observe how physicians performed during moments of communication problem solving in the workplace and if these moments supported the continued improvement of their communication skills through PFL.

Participants had been in practice for 5–32 years (median 13 years) and were pediatricians (n = 2), developmental pediatricians (n = 4), a pediatric orthopedic surgeon (n = 1), and a neonatal intensive care follow-up specialist (n = 1) who provide care for children and youth with developmental disabilities in ambulatory clinics at the hospital. Capability in navigating challenging conversations was felt to be integral to their work yet none of the participants had engaged in formal learning opportunities to enhance their communication skills. The research ethics board from the Bloorview Research Institute approved the conduct of this study. Informed written consent was obtained from each participant.

### Data collection

A research assistant (RA) (LB) with Master’s level training, conducted 25 observations of the eight participants (average 3 observations per participant, range 1 to 4), resulting in over 100 hours of observation over the course of 17 months. We observed physicians as they interacted with patients and families. The RA recorded field notes documenting the general characteristics of the case as well as the physician’s communications and actions. Through these observations, we purposefully identified challenges physicians encountered as they interacted with patients and families to understand how they approached and worked through these situations. For the purposes of this study, we defined ‘challenging conversations’ as those that involved conflict (e.g., differences in opinion between the physician and family), significant emotions (e.g., sadness), or sharing bad news (e.g., new diagnosis, poor prognosis). From our previous research, we anticipated that these moments would require flexible and adaptive communication skills and may stimulate clinicians to seek additional learning to improve their skills [21,29]. Following each observation, we asked participants to reflect on their own perceived challenges during observed interactions with patients and families and how they approached these interactions. Additionally, we conducted post-observation interviews (see Appendix A) to further probe the physician’s thought processes and actions associated with these challenging situations and other challenging conversations that she identified during the observation. At the end of the study, the RA conducted in-depth interviews (see Appendix B) to further understand how they learned in the workplace and how this had evolved over time.

### Data Analysis

Interviews were audiotaped and transcribed. Identifying information was removed from field notes and transcripts. Data was entered into a qualitative data analysis software program (NVivo11, Doncaster) to facilitate the identification and organization of themes. Two team members (AK, LB) independently coded field notes and transcribed interviews as the data was collected. Excerpts that both team members highlighted as ‘challenging conversations’ were shared with the other team members (AO, MZ, MM). Team members then independently coded the excerpts before meeting as a group. Consistent with a constructivist grounded-theory approach [30], participant experiences and researcher perspectives shaped analysis of the data. Themes were identified using constant comparative analysis and further explored through subsequent interviews and observations, with a focus on negative case analysis [31]. Data was analyzed abductively; interpreting the data inductively as it was collected and also deductively by attending to sensitizing concepts from the theoretical framework of adaptive expertise, such as procedural knowledge, conceptual understanding, and PFL [32,33].

Team meetings to analyze and develop the coding framework were held approximately once every eight weeks over the course of 12 months [34]. Dialogue and re-examination of the transcripts and field notes were used to resolve differences in interpretation. Data was collected and analyzed iteratively until the research team deemed that there was sufficient information power to understand the mechanisms underlying PFL in the context of challenging conversations in the pediatric rehabilitation workplace [35]. A final set of codes was applied to the entire data set by one researcher (AK). A detailed audit trail consisting of initial coding, notes from team meetings, and dated versions of the NVivo database was maintained throughout the data collection and analysis process.

## Results

Four interconnected themes that informed our understanding of how physicians continue to learn through challenges in the workplace were identified from our analysis of the interview data and field notes. When engaging in challenging conversations with patients and families, experienced physicians expand their skills through 1) monitoring and attuning to situational and contextual cues, 2) navigating uncertainty, failing, and taking risks while exploring new and varied ways of practicing, and 3) questioning why their strategies were successful or not. Through this 4) constant inquiry and continual investment in problem solving, experienced physicians recognize larger conceptual shifts that generated new approaches for communicating with patients and families.

### Monitoring and attuning to situational and contextual cues

While interacting with patients and their families, participants in this study responded flexibly in the moment dependent on the situation and context. This flexibility requires shifts in perspective; physicians may initially anticipate what may happen during a conversation, but adjust their perspectives to match the needs of each individual patient and family while in the room. This physician describes this flexibility in her communication as ‘constant shifting’ dependent on where the patient and family leads her.

Participant 5: “I feel it’s constantly shifting. I’ll go in with one concept and I’ll come out with a completely different concept because everything that I do is grounded in what the families need or want or what the youth and children need or want at that point in time.”

Experienced physicians attune to multiple cues and strategically probe for information to navigate through these conversations and the many potential possibilities of where to go next. One physician was observed navigating a challenging situation where a teenager with a chronic disability and his mother were in conflict. She purposefully separated the individuals and started by asking about each of their priorities. By attuning to their responses, she then flexibly and strategically approached each conversation:

Participant 5: “I think that helps me understand where the family is at, at *that* specific point in time. Are they anxious? Are they worried? Are they sad? Are they angry? And then, based on those initial impressions, that’s where we pull out the different strategies. Is this going to be a visit where we are more listening? Is this a visit where they’re seeking information? Is this a visit where we need to share information?”

Occasionally, researchers identified patient cues that were not recognized by physicians. In one such observation where an intervention was being offered to a patient, the researcher noticed the mother’s non-committal responses and disinterest in the intervention. The physician however, did not seem to notice these cues and was surprised to hear that the family had declined the intervention when another team member went into the room afterwards to schedule the intervention. In the post-observation interview, the physician voluntarily offers a reflection about this interaction.

Participant 2: “Maybe on reflection, if I’d spent a little bit more time I might have gotten to that they were still scared to move forward with the (intervention)…I didn’t pick up on it then but because they hadn’t gone through with it (the intervention) before, I maybe could have spent a little bit more time probing to see.”

Although the cues were not attended to initially, the patient’s response provided important feedback to the physician, which prompted reflection on what could have been done differently.

### Navigating uncertainty, failing, and taking risks while exploring new and varied ways of practicing

Unexpected responses prompted physicians to pause and search for existing strategies that provided the best fit or to build new strategies. Physicians recognize that the very nature of their practice is unpredictable and it is through these varied situations that they begin to appreciate different perspectives and the many ways of navigating challenging conversations. This physician describes how she has had to respond to unexpected disclosures of abuse and intimate partner violence. In order to respond, she navigates this uncertain territory by pausing, recalibrating, and generating a new, individualized approach.

Participant 1: “I think I give myself time to process that moment in time. And process what I’m seeing and experiencing to see how I have to adapt or change my technique, my approach, my preconception – well, preconceived ideas about the client or family going into that appointment. And once I’ve given myself time then internally I will then change around whatever technique I had preplanned to go in there.”

Navigating uncertainty and trialing new ways of communicating requires effort and can be emotional for physicians. It is not always possible (or feasible) to continually innovate in practice. In this observation, the physician realizes that despite taking time to explain the diagnosis of autism to a parent, her routine process for sharing the diagnosis has failed. When asked if the mother will access the speech therapy recommended for her child, the mother responds by saying, “I think my child is normal and I’m not interested in any more services moving forward.”

In the post-observation interview, this physician explains how sticking to her ‘process’ for sharing a diagnosis of autism helps her to manage the anxiety and fear she experiences when engaging in challenging conversations. However, when this approach fails, it prompts further learning.

Participant 7: “So, learning how to sort of give up control of that (sharing the diagnosis with a routine approach), has been a big part of – of the learning for me. And then to sort of still go with where the parent’s at. So, it’s sort of like, “Okay,” you know, “Let’s find out what you want to do right now. Let’s, you know, figure out how that – how we can make that supportive for your child.”

In navigating challenging conversations, physicians are bound to fail, yet they have learned to be comfortable with failure. They recognize that failure can be valuable in helping them develop and learn new strategies.

Participant 2: “I think I’ve learned a lot through failing initially…I think we learn it over time through failing and not doing it correctly, and through experience and exposure.”

This experienced physician explains that the biggest change in her ability to navigate challenging conversations is that she is “comfortable with being uncomfortable”. Her ability to courageously take risks and try new ways of communicating has expanded her knowledge.

Participant 7: “I think you – as you sort of get into more and more of these situations, you start to trust in your own abilities more to react in the moment. I think, the biggest fear when I started out, was that someone was going to say something and I would like, just not know how to respond.”

Physicians carefully assess the responses and reactions to their varied strategies to determine what works and what doesn’t. By examining this interplay between the success of their strategies and the contexts in which they are used, experts expand their repertoire of communication skills along with conceptual understanding of when and with whom they may be used in the future.

Participant 1: “I think it’s just your style, your swag, your technique that you built over time, right? And I think techniques are built when you’re successful at one thing, so you keep that skill set. When you’re unsuccessful at something you’re like, okay, I’m going to lose this one or maybe that won’t work for this client but work with this family. So I think it’s experience, trial and error, failure, reflecting on that and not taking – like if you had a bad encounter with a parent, not to beat yourself up about it, but say, why was it deemed a bad encounter?…”

Physicians not only monitored the success or failure of their communication strategies but also asked why their strategies were successful or not. This expanded their conceptual understanding of why and how their procedural skills in communication worked in certain contexts.

### Questioning why their strategies were successful or not

When faced with new communication challenges, physicians question why their existing approaches have not been successful within certain contexts thereby expanding their conceptual understanding.

Participant 1: “I think as a clinician you pause to say, okay, this is different. Why is it different? … What was difficult with that encounter?”

However, practice constraints may limit this curiosity and subsequent learning. In one observation, the physician enters a room where the parents seem quite angry. The researcher who has been in the room with the parents throughout the visit, suspects that it is because they have been waiting a long time or because an interaction with one of the team members did not go well. The physician does not directly address the anger and instead moves through the appointment quickly. Afterwards, the physician explains that she chose to make the appointment “brief but effective” due to time constraints.

Participant 5: “Well I guess I chose to not directly address the anger in that last one… But, I could’ve made a choice of saying, “You seem rather upset today…” At that time I made a purposeful choice not to.”

However, when time and space allows, physicians seek an understanding of the differences between their own perspectives and those of their patients and families, prompting them to broaden their conceptual understanding of patient and family perspectives and learn new ways of navigating this challenge in practice. This physician describes how this has evolved over her years in practice. While in the past she might have walked away from a conflict with her patients, she now seeks to better understand the drivers behind why her patients are refusing treatment.

Participant 8: “When I was young and I thought everything was black and white, it was easy. I was like ‘I don’t know, no I wouldn’t recommend that’ and I just would walk away. Now it’s sort of like ‘okay let’s go back to the table, let’s figure out what’s driving this’.”

Through questioning their ways of interacting with patients and families, seeking understanding for why they reacted in the ways they did, and learning from their failures, physicians develop new strategies for communicating. Experts continually reinvest in progressively improving their communication skills and conceptual understanding leading to larger conceptual shifts that transform their practice.

### Constant inquiry and continual investment in problem solving

Experienced physicians in this study are open to new perspectives, engage in constant inquiry, and continually strive for better ways of communicating with patients and families. This physician describes how she feels when faced with a new challenge in her clinical practice.

Participant 1: “I’m excited. It’s strange, because that means that’s a new thought, new idea, new experience for me, and it’s a new learning opportunity. So I’m like whoa, what’s this? I’ve got to learn it.”

These new ways of thinking or shifts in perspective are ubiquitous during clinical encounters. Experienced physicians view this as part of their routine work but identify larger conceptual shifts as more challenging. This physician describes how she can flexibly shift her perspective based on the patient and family’s needs but has had to reconsider how she practices when faced with conceptual shifts that challenge her own beliefs and values and her medical training. In this conversation, the physician references how she is currently grappling with knowing how she will continue to provide care for a child taking immunosuppressive medications and parents adamantly against immunizing their child.

Participant 5: “So those are easy shifts that just automatically happen. But then there are these bigger conceptual shifts where people challenge, kind of, what the general standard is, and those ones are the harder ones.”

Conceptual shifts, described as “shifts of paradigm” and “reconceptualizations of care”, transform how the physician views their work and subsequently alters practice. These larger conceptual shifts in practice have a profound impact on how these physicians approach the work they do. Through these new conceptual understandings, they approach the problem differently and strive to improve their procedural skills and practice through these new perspectives. This physician describes an encounter that prompted a conceptual shift in how she shares a diagnosis of autism with parents. In this situation, the parents disagreed with the diagnosis and as she used all of her usual approaches to help them understand why she felt their child was presenting with autism, she felt that the conversation resulted in her trying to convince the parents that their child was impaired rather than focusing on strengths.

Participant 7: “But here I was, in this situation that I hated being in. And so, after that, I think I was sort of like, “This really didn’t go well. This was not how I wanted to be this kid’s doctor.” And so, “What could I do differently next time?”

Through this and subsequent encounters, she has learned that sometimes she needs to “press pause for a few months and then try again” and that the greatest shift in what she does currently is “going in thinking I’m giving a diagnosis and then walking out not having done so”. Through continually reinvesting in seeking a better understanding of how to approach new challenges, these experienced physicians continue to discover and learn throughout their careers.

## Discussion

We explored *why* some physicians seek improvement and *how* they use the workplace context to build their capabilities. Our results suggest that knowledge building and continual improvement in communication skills are driven by physicians’ inquiry into the challenges of practice; through monitoring and attuning to situational and contextual cues, through taking risks and being comfortable with uncertainty while exploring new and varied ways of practicing, and through seeking why their strategies are successful or not. In this way, experienced physicians continually reinvest cognitive resources in progressive problem solving [28], refining existing knowledge and building new knowledge for navigating communication challenges in practice.

Crucially, our results suggest that PFL in the workplace is driven by an interplay between habits of inquiry and knowledge, inquiry into why certain strategies work and the knowledge required to generate new strategies when needed. As their conceptual understanding grows, physicians are prepared for future learning, progressively solving problems of practice that are at the edge of their competence. Thus, these physicians actively engaged in determining what and how they learned from clinical encounters through continual and effortful engagement with their work [28,36].

However, physicians may not always rise to the challenge of improving their communication skills, even though they demonstrate the capacity to do so in other situations. Sometimes they may not recognize the cues that a new strategy is needed or they choose to apply their routine efficiencies inappropriately, resulting in failures. They may also be constrained by time or their own assumptions about what patients and families need. It is not always possible or feasible to recognize and take advantage of every learning opportunity. As others have noted, “Learning in areas of weakness is, by definition, difficult” [8]. While this study explored mechanisms for why some physicians take the opportunity generate new knowledge, future studies focusing on when physicians miss opportunities or purposefully choose not to engage in learning will be important for understanding the effects of practice constraints, emotional safety, and other restrictions on PFL.

Our results extend those from a recent study examining how experienced physicians used learning cues from clinical encounters in the workplace to improve communication skills through reflection and deliberate fine-tuning of their repertoires [25]. In our study, physicians not only learned by drawing upon the cues in the workplace, they *actively* generated new approaches for communicating (procedural knowledge) linked to a deep understanding of why these strategies work in clinical practice (conceptual understanding). Inquiry pushed these experienced physicians to think ‘outside-the-box’, to go beyond their routine efficiencies to innovate as they work and provide care. Habits of inquiry appear essential to drive the continual reinvestment in learning that enables physicians to tackle progressively more complex representations of recurrent problems [28]. These results reinforce those of a study examining the development of adaptive expertise in geriatric medicine where curiosity motivated clinicians to strive for continual learning [37].

Moreover, these experienced physicians were able to explore new ways of practicing through their “comfort with feeling uncomfortable” [38] and their willingness to fail. Thus, by feeling safe to take risks with trialing new strategies in a variety of situations, having the freedom to experiment, and engaging in the feedback from their interactions with children and families, these experiences enriched and deepened their conceptual understanding. This type of learning, which is tied to content knowledge and embedded within context, enabled direct transfer to work activities unlike formal CPD activities that are often disconnected from the actual work context [39,40].

Our results provide empirical evidence for a *mechanism* through which physicians actively learn from the workplace context. It appears that habits of inquiry, alongside conceptual knowledge [41], are crucial to PFL. This better understanding of ‘how’ and ‘why’ habits of inquiry are activated can guide CPD efforts and medical education that aims to foster inquiry-based approaches for learning in the workplace [33]. Formal CPD has been criticized as being a ‘tick-box’ activity where learning occurs in settings separate from the context of practice [42,43]. This study demonstrates how PFL occurs in practice and through our daily work [8]. These embedded learning opportunities invoked practice-changing conceptual shifts that went beyond simple understanding of communication strategies or demonstration of communication skills typical of formal CPD outcomes. Patient cues and responses in challenging conversations provided critical feedback for informed self-assessment that promoted reflection and generation of new knowledge [44]. This is particularly important, as previous research suggests that physicians experience tension when the feedback received is incongruent with their self-appraisal of their performance [45]. However, physicians in this study navigated this tension effectively, questioning how they could improve when patient cues suggested a different approach was required.

PFL as an unplanned yet intentional form of learning in the workplace *is* CPD [44]. Recognizing it as such challenges our current notions of what constitutes CPD and the methods for documenting maintenance of certification. Additionally, it urges us as educators to consider how we can support PFL through fostering inquiry-based approaches for learning in the workplace. As learners engage in challenging conversations, educators may support future learning through inquiring why an approach worked, or why it did not, and how the context (e.g. for this patient in this family) may have influenced the response [46]. Our findings support creation of an educational environment that allows the learner to seek help when they are feeling uncomfortable [47] and encourages them to develop comfort with uncertainty [48,49]. Educators who entrust learners to take appropriate risks [29,50] will allow them to reap the benefits of seeing how the natural variation in practice enhances their understanding of why certain strategies work in certain situations and not others. These key elements of supporting conceptual understanding, experimentation with new and innovative strategies, and learning from natural variation are known to support the development of adaptive expertise and can be promoted through enhancing habits of inquiry [32].

We do not know if our results apply to other areas of medicine or different cultural contexts. While communication is central to the practice of medicine, the nature of challenging conversations within developmental pediatrics may be different from those of other fields. For example, in this area of medicine, when a family ‘rejects’ a diagnosis, this is rarely the end of care. Rather, maintaining relationships with the patient and family may allow the physician to later bring back concerns and revisit diagnoses and interventions. Thus, the findings generated from this study may have differing effects in other areas of medicine. Our study was conducted at a North American tertiary care academic pediatric rehabilitation hospital that values patient and family-centred care. This cultural context may have influenced our results and therefore further research examining the intersection of communication, context, and habits of inquiry is still needed. The participants in this study demonstrated habits of inquiry to improve their communication skills and to learn, however, our study did not explore why these individuals were driven towards these activities or how to promote habits of inquiry in experienced physicians.

Inquiry motivates physicians to improve their ability to navigate challenging conversations, enabling them to generate new knowledge as they provide care to patients and families. This interplay between habits of inquiry and knowledge explains why some physicians hone their communication skills through this informal yet intentional learning in practice. However, time constraints, assumptions regarding the needs of patients and families, and the emotional demands of challenging conversations may limit physicians’ abilities to fully engage in PFL. Future studies that explore how educators can nurture and grow these habits of inquiry in learners and how to create workplace-learning environments that address practice constraints for PFL may enable us to better support CPD and lifelong learning.

## Appendix A – Semi-structured post-observation interview guide

1. Is there anything from today’s observation that stands out to you, in terms of your interactions with the clients and families?
2. Was there anything that occurred during today’s interactions with your clients and families that was unexpected?
3. How did you approach the clinical interactions today based on information provided in the health records chart, prior to entering the room? Or after information was gathered during the interaction?
4. Were there specific cues (or relevant pieces of information) from the family that you noticed during the encounter? Why were they important? What happened when you heard this information?
5. Were there any cues that you identified, but did not specifically address in this encounter? Why?
6. During today’s observation, I noticed ___________ (description of challenging conversation). Could you tell me what you were thinking during that part of the interaction?

## Appendix B – Semi-structured exit interview guide

1. Can you briefly describe the type of work you do at X hospital and how long you have been in practice?
2. In your work, you see clients and families who come to you with many different needs, goals, and beliefs. How do you learn about these different needs/goals/beliefs? What happens if they are different from your perspective or the team’s perspective? How do you approach this?
3. Sometimes we meet resistance, or even conflict, with clients and families while providing care. Can you give me an example of this from your work setting? How did you approach this?
4. At times, physicians need to shift their own perspectives to provide care that is attuned to the needs/goals/beliefs of their clients and families. We call these adjustments “shifts”. Do you think that you make “shifts” when interacting with clients/families? If so, could you describe a time when this occurred? How did this shift affect the care of your client? Did this shift influence how you approach your clients in other cases?
5. How do you recognize when you need to make a “shift”?
6. How do you feel you respond when you recognize that a “shift” in your own perspective is needed?
7. How did you learn to recognize and/or respond to “shifts”?
8. Are there specific skills that you think are important to be effective at being able to recognize and/or respond to “shifts”? How did you learn to develop these skills?
9. With respect to “shifts”, are there things you do differently now that you did not do five or 10 years ago?
10. Do you teach students to think about “shifts”? How do you teach this to trainees?

## References

[1] Mylopoulos M, Lohfeld L, Norman GR, Dhaliwal G, Eva KW. Renowned physicians’ perceptions of expert diagnostic practice. Acad Med. 2012; 87(10): 1413–7. DOI: 10.1097/ACM.0b013e31826735fc 22914510

[2] Mylopoulos M, Farhat W. “I can do better”: exploring purposeful improvement in daily clinical work. Adv Health Sci Educ. 2015; 20(2): 371–83. DOI: 10.1007/s10459-014-9533-5 25108367

[3] Law M, Leung P, Veinot P, Miller D, Mylopoulos M. A Qualitative Study of the Experiences and Factors That Led Physicians to Be Lifelong Health Advocates. Acad Med. 2016; 91(10): 1392–7. DOI: 10.1097/ACM.0000000000001316 27438157 PMC5044810

[4] Kawamura AA, Orsino A, Mylopoulos M. Integrating competencies: exploring complex problem solving through case formulation in developmental pediatrics. Acad Med. 2014; 89(11): 1497–501. DOI: 10.1097/ACM.0000000000000475 25250750

[5] Mylopoulos M, Kulasegaram K, Woods NN. Developing the experts we need: Fostering adaptive expertise through education. J Eval Clin Pract. 2018; 24(3): 674–7. DOI: 10.1111/jep.12905 29516651

[6] Bransford JD, Schwartz DL. Chapter 3: Rethinking Transfer: A Simple Proposal With Multiple Implications. Review of research in education. 1999; 24(1): 61–100. DOI: 10.3102/0091732X024001061

[7] Mylopoulos M, Brydges R, Woods NN, Manzone J, Schwartz DL. Preparation for future learning: a missing competency in health professions education? Med Educ. 2016; 50(1): 115–23. DOI: 10.1111/medu.12893 26695471

[8] Regehr G, Mylopoulos M. Maintaining competence in the field: learning about practice, through practice, in practice. The Journal of continuing education in the health professions. 2008; 28: S19–23. DOI: 10.1002/chp.203 19058249

[9] Mylopoulos M, Scardamalia M. Doctors’ perspectives on their innovations in daily practice: implications for knowledge building in health care. Med Educ. 2008; 42(10): 975–81. DOI: 10.1111/j.1365-2923.2008.03153.x 18823516

[10] Teunissen PW, Scheele F, Scherpbier AJ, et al. How residents learn: qualitative evidence for the pivotal role of clinical activities. Med Educ. 2007; 41(8): 763–70. DOI: 10.1111/j.1365-2923.2007.02778.x 17661884

[11] Berkhout JJ, Helmich E, Teunissen PW, van der Vleuten CPM, Jaarsma ADC. Context matters when striving to promote active and lifelong learning in medical education. Med Educ. 2018; 52(1): 34–44. DOI: 10.1111/medu.13463 28984375

[12] ten Cate OTJ, Kusurkar RA, Williams GC. How self-determination theory can assist our understanding of the teaching and learning processes in medical education. AMEE Guide No. 59. Med Teach. 2011; 33(12): 961–73. DOI: 10.3109/0142159X.2011.595435 22225433

[13] Artino AR Jr., It’s Not All in Your Head: Viewing Graduate Medical Education Through the Lens of Situated Cognition. J Grad Med Educ. 2013; 5(2): 177–9. DOI: 10.4300/JGME-D-13-00059.1 24404254 PMC3693674

[14] Evensen DH, Salisbury-Glennon JD, Glenn J. A Qualitative Study of Six Medical Students in a Problem-Based Curriculum: Toward a Situated Model of Self-Regulation. J Educ Psych. 2001; 93(4): 659–76. DOI: 10.1037/0022-0663.93.4.659

[15] Moulton C, Regehr G, Lingard L, Merritt C, MacRae H. ‘Slowing Down When You Should’: Initiators and Influences of the Transition from the Routine to the Effortful. J Gastro Surg. 2010; 14(6): 1019–26. DOI: 10.1007/s11605-010-1178-y 20309647

[16] Steenhof N, Woods NN, Mylopoulos M. Exploring why we learn from productive failure: insights from the cognitive and learning sciences. Adv Health Sci Educ. 2020; 25(5): 1099–106. DOI: 10.1007/s10459-020-10013-y 33180211

[17] Woods NN. Science is fundamental: the role of biomedical knowledge in clinical reasoning. Med Educ. 2007; 41(12): 1173–7. DOI: 10.1111/j.1365-2923.2007.02911.x 18045369

[18] Woods NN, Brooks LR, Norman GR. It All Make Sense: Biomedical Knowledge, Causal Connections and Memory in the Novice Diagnostician. Adv Health Sci Educ. 2007; 12(4): 405–15. DOI: 10.1007/s10459-006-9055-x 17318360

[19] Baghdady MT, Carnahan H, Lam EWN, Woods NN. Integration of Basic Sciences and Clinical Sciences in Oral Radiology Education for Dental Students. J Dental Educ. 2013; 77(6): 757–63. DOI: 10.1002/j.0022-0337.2013.77.6.tb05527.x 23740912

[20] Mylopoulos M, Woods N. Preparing medical students for future learning using basic science instruction. Med Educ. 2014; 48(7): 667–73. DOI: 10.1111/medu.12426 24909528

[21] Kawamura A, Harris I, Thomas K, Mema B, Mylopoulos M. Exploring How Pediatric Residents Develop Adaptive Expertise in Communication: The Importance of “Shifts” in Understanding Patient and Family Perspectives. Acad Med. 2020; 95(7): 1066–72. DOI: 10.1097/ACM.0000000000002963 31464732

[22] Ha JF, Longnecker N. Doctor-patient communication: a review. The Ochsner journal. 2010; 10(1): 38–43. 21603354 PMC3096184

[23] Vogel L. Patient complaints about Canadian doctors on the rise. Can Med Assoc J. 2018; 190(13): E408–E. DOI: 10.1503/cmaj.109-5585 29615429 PMC5880654

[24] Silverman J. Clinical communication training in continuing medical education: possible, do-able and done? Patient Educ Couns. 2011; 84(2): 141–2. DOI: 10.1016/j.pec.2011.05.026 21696909

[25] Sehlbach C, Teunissen PW, Driessen EW, et al. Learning in the workplace: Use of informal feedback cues in doctor-patient communication. Med Educ. 2020; 54(9): 811–20. DOI: 10.1111/medu.14148 32150761 PMC7496915

[26] Eva KW. On the generality of specificity. Med Educ. 2003; 37(7): 587–8. DOI: 10.1046/j.1365-2923.2003.01563.x 12834414

[27] Mylopoulos M, Woods NN. When I say … adaptive expertise. Med Educ. 2017; 51(7): 685–6. DOI: 10.1111/medu.13247 28224707

[28] Bereiter C, Scardamalia M. Surpassing Ourselves: An Inquiry into the Nature and Implications of Expertise. Chicago, IL: Open Court; 1993.

[29] Lynch J, Orsino A, Kawamura A. Productive struggle and failing safely: implications for developing adaptive expertise in communication. Adv Health Sci Educ. 2022; 27(5): 1331–44. DOI: 10.1007/s10459-022-10175-x 36334228

[30] Charmaz K. Shifting the grounds: Constructivist grounded theory methods. In: Morse JM, Stern PN, Corbin J, Bowers B, Charmaz K, Clarke AE (eds.), Developing grounded theory: The second generation. 2009; 127–54. Walnut Creek: Left Coast Press.

[31] Charmaz K. The legacy of Ansel Strauss in constructivist grounded theory. Stud Symbol Interact. 2008; 32: 127–41. DOI: 10.1016/S0163-2396(08)32010-9

[32] Hatano G, Inagaki K. Two courses of expertise. In: Stevenson H, Azuma H, Hakuta K (eds.), Child Development and Education in Japan. 1986; 27–36. New York: W H Freeman.

[33] Valbuena G, O’Brien B, Ten Cate O, O’Sullivan P. Inquiry in the Medical Curriculum: A Pedagogical Conundrum and a Proposed Solution. Acad Med. 2019; 94(6): 804–8. DOI: 10.1097/ACM.0000000000002671 30920445

[34] Morse JM. Critical analysis of strategiesfor determining rigor in qualitative inquiry. Qual Health Res. 2015; 25: 1212–22. DOI: 10.1177/1049732315588501 26184336

[35] Malterud K, Siersma VD, Guassora AD. Sample Size in Qualitative Interview Studies: Guided by Information Power. Qual Health Res. 2016; 26(13): 1753–60. DOI: 10.1177/1049732315617444 26613970

[36] Watling C, Ginsburg S, LaDonna K, Lingard L, Field E. Going against the grain: An exploration of agency in medical learning. Med Educ. 2021; 55(8): 942–50. DOI: 10.1111/medu.14532 33780013

[37] Kua J, Teo W, Lim WS. Learning experiences of adaptive experts: a reflexive thematic analysis. Adv Health Sci Educ. 2022; 27(5): 1345–59. DOI: 10.1007/s10459-022-10166-y PMC9859887 36626011

[38] Ilgen JS, Eva KW, de Bruin A, Cook DA, Regehr G. Comfort with uncertainty: reframing our conceptions of how clinicians navigate complex clinical situations. Adv Health Sci Educ. 2019; 24(4): 797–809. DOI: 10.1007/s10459-018-9859-5 30390181

[39] McMahon GT. Inspiring Curiosity and Restoring Humility: The Evolution of Competency-Based Continuing Medical Education. Acad Med. 2018; 93(12): 1757–9. DOI: 10.1097/ACM.0000000000002339 29952769

[40] Teunissen PW, Dornan T. Lifelong learning at work. BMJ. 2008; 336(7645): 667–9. DOI: 10.1136/bmj.39434.601690.AD 18356236 PMC2270942

[41] Forsey J, Ng S, Rowland P, Freeman R, Li C, Woods NN. The Basic Science of Patient–Physician Communication: A Critical Scoping Review. Acad Med. 2021; 96(11S): S109–S18. DOI: 10.1097/ACM.0000000000004323 34348382

[42] Sargeant J, Wong BM, Campbell CM. CPD of the future: a partnership between quality improvement and competency-based education. Med Educ. 2018; 52(1): 125–35. DOI: 10.1111/medu.13407 28984354

[43] Horsley T, Moreau K, Lockyer J, Zeiter J, Varpio L, Campbell C. More Than Reducing Complexity: Canadian Specialists’ Views of the Royal College’s Maintenance of Certification Framework and Program. J Cont Educ Health Prof. 2016; 36(3): 157–63. DOI: 10.1097/CEH.0000000000000099 27583991

[44] Sargeant J, Mann K, Sinclair D, Ferrier S, Muirhead P, van der Vleuten C, et al. Learning in practice: experiences and perceptions of high-scoring physicians. Acad Med. 2006; 81(7): 655–60. DOI: 10.1097/01.ACM.0000232422.81299.b7 16799293

[45] Mann K, Vleuten CPMvd, Eva K, Armson H, Chesluk B, Dornan T, et al. Tensions in informed self-assessment: how the desire for feedback and reticence to collect and use it can conflict. Acad Med. 2011; 86(9): 1120–7. DOI: 10.1097/ACM.0b013e318226abdd 21785309

[46] Dornan T, Boshuizen H, King N, Scherpbier A. Experience-based learning: a model linking the processes and outcomes of medical students’ workplace learning. Med Educ. 2007; 41(1): 84–91. DOI: 10.1111/j.1365-2929.2006.02652.x 17209896

[47] Ilgen JS, de Bruin ABH, Teunissen PW, Sherbino J, Regehr G. Supported Independence: The Role of Supervision to Help Trainees Manage Uncertainty. Acad Med. 2021; 96(11S): S81–S6. DOI: 10.1097/ACM.0000000000004308 34348381

[48] Ilgen JS, Bowen JL, de Bruin ABH, Regehr G, Teunissen PW. “I Was Worried About the Patient, but I Wasn’t Feeling Worried”: How Physicians Judge Their Comfort in Settings of Uncertainty. Acad Med. 2020; 95(11S): S67–S72. DOI: 10.1097/ACM.0000000000003634 32769464

[49] Ilgen JS, Teunissen PW, de Bruin ABH, Bowen JL, Regehr G. Warning bells: How clinicians leverage their discomfort to manage moments of uncertainty. Med Educ; 2020. DOI: 10.1111/medu.14304 32748479

[50] Ten Cate O, Carraccio C, Damodaran A, Gofton W, Hamstra SJ, Hart DE, et al. Entrustment Decision Making: Extending Miller’s Pyramid. Acad Med. 2021; 96(2): 199–204. DOI: 10.1097/ACM.0000000000003800 33060399

